# Comparative analysis of Australian hospital antimicrobial utilization, using the WHO AWaRe classification system and the adapted Australian Priority Antimicrobial List (PAL)

**DOI:** 10.1093/jacamr/dlab017

**Published:** 2021-02-27

**Authors:** Nadine T Hillock, Erin Connor, Courtenay Wilson, Brendan Kennedy

**Affiliations:** 1 Communicable Disease Control Branch, Department for Health and Wellbeing, Adelaide, SA, 5000, Australia; 2 Infectious Diseases Department, Royal Adelaide Hospital, Adelaide, SA, 5000, Australia

## Abstract

**Background:**

In 2020 the Australian Priority Antibacterial List (PAL) was developed to support national surveillance of antibacterial usage.

**Objectives:**

To compare the WHO AwaRe classification system with the Australian PAL to analyse antibacterial utilization in Australian acute care hospitals.

**Methods:**

Monthly antibacterial usage rates (defined daily dose per 1000 occupied bed days) were calculated using pharmacy dispensing records together with patient occupancy data for all acute care hospitals contributing to the National Antimicrobial Utilisation Surveillance Program for 2015–19. Annual usage rates as a proportion were determined using the WHO AWaRe and Australian PAL categorization systems.

**Results:**

In 2019, 70.0% of total-hospital aggregate antibacterial use in Australian acute-care hospitals fell into the WHO *Access* category, with 29.4% of usage in *Watch* and 0.6% in the *Reserve* category. Analysis using the PAL classification system showed 40.1% of hospital usage fell into the *Access* category, 55.6% in *Curb* and 3.8% in the *Contain* categories. On average, cefazolin usage comprised 12.5% of acute hospital usage.

**Conclusions:**

Cefazolin, a first-line agent for surgical prophylaxis in Australia, was identified as a key antibacterial driving the differing results seen between the two classification systems. Data on the proportions of day surgery relative to inpatient surgical cases would assist the accuracy of benchmarking usage between hospitals using the PAL categorization system. The use of a targeted, nationally approved prioritized classification system can provide a focus for antimicrobial stewardship at a national level, however a clear understanding of the consumption metric used, as well as its limitations, are required for interpretation.

## Introduction

In 2017, the WHO updated its Model List of Essential Medicines, and incorporated a classification system for antibacterials, the Access, Watch, Reserve (AWaRe) categories (Table S1, available as Supplementary data at *JAC-AMR* Online).^1^ Development of AWaRe was based on an assessment of the most frequent and severe bacterial infections worldwide, with the aim of improving access and clinical outcomes, while reducing antimicrobial resistance and preserving the effectiveness of last-line antibiotics.^2^

The AWaRe framework may be used by countries to set targets for antimicrobial stewardship (AMS) initiatives.^3^ The WHO target is for the proportion of global human antibacterial consumption in the *Access* category to be at least 60% by 2023, with a consequent reduction in the relative use of antibacterials in the other categories.^4^^,^^5^

A number of countries have utilized or adapted the AWaRe classifications for their own AMS strategies.^6–10^ In 2020, the Priority Antibacterial List (PAL) for systemic antibacterials was developed and published by the Australian Commission on Safety and Quality in Health Care (ACSQHC).^11^ The PAL categorization, adapted from the AWaRe classification system, aimed to increase the relevance to the Australian setting by assigning antibacterials to a category based on whether or not they were recommended in nationally endorsed guidelines as a first-line treatment.^12^ Other considerations included the risks of antimicrobial resistance (AMR) or healthcare-associated infections (HAIs) in human health based on expert consensus.^13^

Although the WHO has set a target for *Access* antibacterial consumption, there is currently no endorsed goal for the Australian-adapted PAL. Rather, it is recommended that the PAL be used locally to monitor proportions of antibacterial use over time, with the aim of increasing the proportion of use in the PAL *Access* category and using the tool to educate prescribers and limit use in the *Contain* category.^11^

The Australian National Antimicrobial Utilisation Surveillance Program (NAUSP) is a voluntary initiative supporting AMS programmes in Australian acute-care public and private hospitals.^14^ Hospitals submit monthly antibacterial usage data (dispensing and ward distribution) for acute adult inpatients (volume-based surveillance) to NAUSP via an online portal. This study analyses antibacterial utilization in Australian acute-care hospitals contributing to NAUSP over the 5 year period 2015–19, comparing the WHO AWaRe classification system with the adapted Australian classification system. In addition, usage in each PAL category across all Australian public Principal Referral hospitals was compared to understand which antibacterials are driving the trends in proportions of usage.

## Methods

Antibacterials included in the NAUSP dataset were tabulated and their assignment into the WHO AWaRe and the Australian PAL categories were compared (Tables S1 and S2). Antibacterial usage data from 214 Australian acute care hospitals, derived from pharmacy dispensing records, were converted from total quantity (grams) into total Defined Daily Doses (DDDs) using the WHO-ATC Index definition of the assumed average adult maintenance dose.^15^ Topical antibacterial formulations were excluded. Monthly aggregate antibacterial usage rates [DDDs per 1000 Occupied Bed Days (OBDs)] were calculated for Australian acute-care hospitals contributing to NAUSP for the 5 year period 2015–19.

Usage rates were then calculated according to AWaRe and PAL classifications as a proportion of total use. 209 hospitals were included in the analysis, as they had contributed at least six consecutive months of numerator and denominator data in the study period. All data were extracted from the NAUSP database on the 28 May 2020.

This study was considered negligible risk research and met the conditions for exemption from ethics review as all hospitals were de-identified, and no data on prescribers or consumers were accessed.

## Results

**Figure 1.**
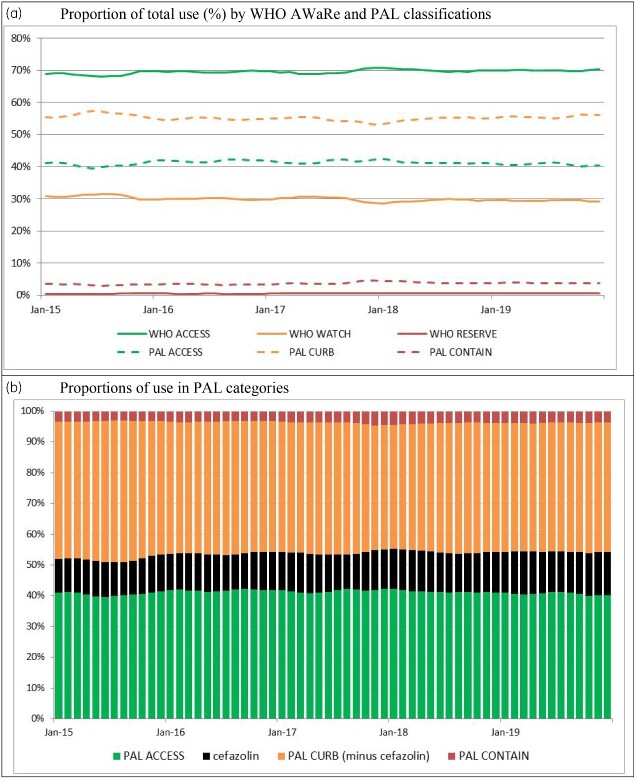
Antibacterial usage in hospitals contributing to the NAUSP program. (a) Proportion of total use (%) by WHO AWaRe and PAL classifications. (b) Proportions of use in PAL categories, showing cefazolin usage as a driver of *Curb*.

Figure 1(a) shows the proportions of usage of categorized antibiotics in Australian hospitals for the period 2015–19 using both the AWaRe and PAL classifications, illustrated as a proportion of total use. Figure 1(b) highlights the usage of cefazolin as a driver of *Curb* usage in the PAL.

When cefazolin is included in the *Curb* category, the proportion of acute hospital antibacterial use in the *Access* category falls to a monthly mean over 60 months of 41.2% (SD: 0.7%). If cefazolin was to be included in the *Access* category (as in the WHO AWaRe system), the mean monthly proportion of use in the *Access* category would increase to 53.6% (SD: 1.1%).

Due to the differences in the WHO and Australian classification systems (Table S2), on average, 28.4% of monthly antibacterial usage shifts from WHO *Access* to PAL *Curb* or *Contain* categories. Cefazolin is the predominant driver, with cefazolin usage constituting 43.8% of this difference on average per month. The mean monthly aggregate usage rate over 60 months for cefazolin for all included contributors was 106.6 DDD/1000 OBDs. On average, this correlated to 12.5% of total monthly antibacterial usage.

Amoxicillin/clavulanic acid and cefalexin are also key antibacterials contributing to the differing proportions of usage between the WHO and PAL *Access* classifications, and to a lesser extent, clindamycin, amikacin and cefalotin.

**Figure 2.**
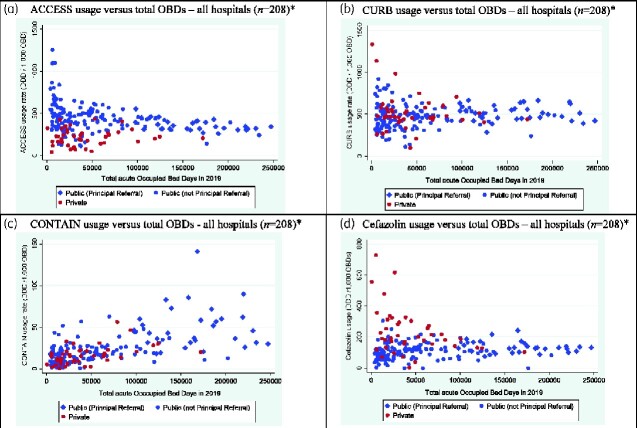
Annual antibacterial use per hospital for 2019 for the PAL categories and cefazolin versus total acute hospital occupied bed days (OBDs). *One public Group D hospital excluded—extreme outlier (small day surgical hospital).

NAUSP data includes 100% representation from Australian public hospitals categorized by the Australian Institute of Health and Welfare (AIHW) as Principal Referral hospitals.^16^ Analysis of usage by PAL category in the Principal Referral hospitals illustrated a marked variation in cefazolin usage (Figure S1); the mean usage rate in 2019 across the 31 hospitals was 131.6 DDD/1000 OBDs (SD: 38.7). Acute OBDs in 2019 varied substantially between the Principal Referral hospitals, with the median being 164 621 (IQR: 125 450–192 235). The variance in the cefazolin usage rate decreased as the number of OBDs increased (Figure 2d).

This trend was seen across all 209 contributor hospitals. The variation in usage rates of *Access* and *Curb* antibacterials is greatest when OBDs are lower, with the variance decreasing as OBDs increased. For *Contain* antibacterials, usage increased as OBDs increased. Figure 2(a–c) illustrates the variation in the *Contain*, *Curb* and *Access* antibacterial usage rates respectively by total acute OBDs, differentiated by private and public facilities.

## Discussion

The WHO target of at least 60% of human consumption being classified as AWaRe *Access* is a goal across all healthcare sectors. These analyses used hospital inpatient consumption data, therefore it is to be expected that there would be a higher proportion of *Watch* and *Reserve* agents used in this acute setting than across all Australian healthcare sectors combined. A study of antibacterial use in the UK using an England-adapted AWaRe index found that although the volume of antibacterial use was higher in the primary care setting, the proportion of *Watch* and *Reserve* agents was higher in the secondary care setting.^7^ In 2016, 49.7% of antibacterial use in secondary care in the UK was classified as *Access*.^7^ In our study, 70.0% of acute Australian hospital use over 5 years was classified as *Access* using the WHO AWaRe classification system, however using the Australian PAL categorization only 40.1% of usage was in the *Access* category. Although there is currently no agreed target for the Australian-adapted PAL, these results illustrate the importance of setting-specific goals if and when proportion targets are determined.

In most instances where there were differences between the AWaRe and PAL classifications, the PAL was more restrictive than AWaRe (Table S2). This discrepancy was primarily driven by use of cefazolin, which classified as *Access* in AWaRe but as *Curb* in the PAL. While surgical prophylaxis is the main indication for cefazolin use in Australia, it is also considered a second-line agent for treatment of some infections, for example soft tissue infections such as cellulitis.^12^ Recent Australian surgical audits found that 37.4% of cefazolin doses used for surgical prophylaxis were considered inappropriate, with post-procedural doses being a common reason for inappropriate use.^17^ Although population level surveillance may not provide indication-specific differentiation, inclusion of cefazolin in *Curb* allows it to be a focus antibacterial for Australian hospitals and policymakers to target stewardship interventions.

Similarly, although cefalexin and amoxicillin/clavulanic acid are classified as *Access* by WHO, because they are known to be over-prescribed in Australian clinical practice^18^ and are a focus area for stewardship, they are classified as *Curb* in the PAL.

The varying proportions of use of cefazolin between Principal Referral hospitals illustrates that usage rates for non-*Access* antibacterials must be interpreted with caution. Although Principal Referral hospitals have patients with similarly acute illness, they may differ with regards to specialty care and the admitted patient activity, and rates of day surgical cases. When used for benchmarking, the metric used for Australian hospital surveillance (DDD/1000 OBDs)^19^ does not allow for the comparative difference in the proportions of day surgeries (where no OBD is recorded). For smaller hospitals with lower OBD counts and a higher proportion of day surgery, the impact on usage rates may be more marked. As illustrated in Figure 2(b), hospitals with lower total OBDs showed a greater variance in the usage of antibacterials in the *Curb* category.

### Limitations

This study is limited to hospital surveillance data captured by the NAUSP. All Australian Principal Referral hospitals and the majority of larger private and public hospitals are included, however, participation by smaller rural and remote hospitals is less than 50%. NAUSP surveillance data is limited to acute-hospital adult inpatient usage.

### Conclusions

The use of a targeted and nationally adapted prioritized classification system can help countries provide a focus for AMS at a national level. However, a clear understanding of the consumption metric used, as well its limitations, are required for accurate interpretation. Country-specific categorization tools can assist hospitals in analysing their usage data to inform antimicrobial restriction policies and assess the outcomes of stewardship initiatives at a hospital, state or national level. This analysis illustrates that local adaptation may markedly change the proportions of use of each category, which may also be impacted by the dataset inclusions and exclusions, as well as the metric used to quantify use. The setting of targets for usage proportions in each PAL category will require consideration of the dataset population (e.g. hospital or community setting), the acuteness of the healthcare setting, and the type of numerator and denominator employed to measure usage.

## Supplementary Material

dlab017_Supplementary_Data
