# Supplementary material for: Comparative analysis of Australian hospital antimicrobial utilization, using the WHO AWaRe classification system and the adapted Australian Priority Antimicrobial List (PAL)
Source: JAC Antimicrob Resist. 2021 Feb 27;3(1):dlab017. doi: 10.1093/jacamr/dlab017 (PMC8210107; doi:10.1093/jacamr/dlab017)
Supplement: dlab017_Supplementary_Data [file dlab017_supplementary_data.docx]

**Supplementary data**

**Table S1: Antimicrobials used in Australian hospitals by AWaRe and PAL classifications**

| **Antibacterial** | **Australian PAL** | **WHO AWaRe** |
| --- | --- | --- |
| amikacin | Contain | Access |
| amoxicillin | Access | Access |
| amoxicillin-clavulanic acid | Curb | Access |
| ampicillin | Access | Access |
| azithromycin | Curb | Watch |
| aztreonam | Contain | Reserve |
| benzathine benzylpenicillin | Access | Access |
| benzylpenicillin | Access | Access |
| ceftazidime-avibactam | Contain | Reserve |
| cefaclor | Curb | Watch |
| cefalexin | Curb | Access |
| cefalothin | Curb | Access |
| cefazolin | Curb | Access |
| cefepime | Contain | Watch |
| cefotaxime | Curb | Watch |
| cefoxitin | Curb | Watch |
| ceftaroline | Contain | Watch |
| ceftazidime | Contain | Watch |
| ceftolozane-tazobactam | Contain | Reserve |
| ceftriaxone | Curb | Watch |
| cefuroxime | Curb | Watch |
| chloramphenicol | Access | Access |
| ciprofloxacin | Curb | Watch |
| clarithromycin | Curb | Watch |
| clindamycin | Curb | Access |
| colistin | Contain | Reserve |
| daptomycin | Contain | Reserve |
| dicloxacillin | Access | Access |
| doripenem | Contain | Watch |
| doxycycline | Access | Access |
| ertapenem | Contain | Watch |
| erythromycin | Curb | Watch |
| fidaxomicin | Curb | Not categorised |
| flucloxacillin | Access | Access |
| fosfomycin (oral) | Contain | Watch |
| gentamicin | Access | Access |
| imipenem-cilastatin | Contain | Watch |
| lincomycin | Curb | Watch |
| linezolid | Contain | Reserve |
| meropenem | Contain | Watch |
| metronidazole | Access | Access |
| minocycline (oral) | Access | Watch |
| moxifloxacin | Contain | Watch |
| nitrofurantoin | Access | Access |
| norfloxacin | Curb | Watch |
| phenoxymethylpenicillin | Access | Access |
| piperacillin-tazobactam | Curb | Watch |
| pivmecillinam | Contain | Access |
| polymixin B | Contain | Reserve |
| pristinamycin | Contain | Watch |
| procaine benzylpenicillin | Access | Access |
| rifaximin | Curb | Watch |
| rifampicin | Curb | Watch |
| roxithromycin | Curb | Watch |
| sodium fucidate (Fusidic acid) | Curb | Watch |
| spiramycin | Curb | Watch |
| streptomycin | Access | Watch |
| sulfamethoxazole-trimethoprim | Access | Access |
| teicoplanin | Curb | Watch |
| tetracycline | Access | Access |
| tigecycline | Contain | Reserve |
| tinidazole | Access | Not categorised |
| tobramycin | Access | Watch |
| trimethoprim | Access | Access |
| vancomycin | Curb | Watch |

**Table S2: Alignment in categorisation: AWaRe versus PAL**

|  | | **AUSTRALIAN PAL CATEGORY** | | |
| --- | --- | --- | --- | --- |
|  |  | **ACCESS (20)** | **CURB (25)** | **CONTAIN (20)** |
| **WHO AWARE** | **ACCESS (23)** | amoxicillin  ampicillin  benzathine benzylpenicillin  benzylpenicillin  chloramphenicol  dicloxacillin  doxycycline  flucloxacillin  gentamicin  metronidazole  nitrofurantoin  phenoxymethylpenicillin  procaine benzylpenicillin  sulfamethoxazole-trimethoprim  tetracycline  trimethoprim | ***amoxicillin-clavulanic acid***  ***cefalexin***  ***cefalothin***  ***cefazolin***  ***clindamycin*** | ***amikacin***  ***pivmecillinam*** |
|  | **WATCH (32)** | ***minocycline (oral)***  ***streptomycin***  ***tobramycin*** | azithromycin  cefaclor  cefotaxime  cefoxitin  ceftriaxone  cefuroxime  ciprofloxacin  clarithromycin  erythromycin  lincomycin  norfloxacin  piperacillin-tazobactam  rifaximin  rifampicin  roxithromycin  fusidic acid  spiramycin  teicoplanin  vancomycin | ***cefepime***  ***ceftaroline***  ***ceftazidime***  ***doripenem***  ***ertapenem***  ***fosfomycin (oral)***  ***imipenem-cilastatin***  ***meropenem***  ***moxifloxacin***  ***pristinamycin*** |
|  | **RESERVE (8)** |  |  | aztreonam  ceftazidime-avibactam  ceftolozane-tazobactam  colistin  daptomycin  linezolid  polymyxin B  tigecycline |
|  | **NOT CATEGORISED**  **(2)** | ***tinidazole*** | ***fidaxomicin*** |  |

**Figure S1. Proportionate antibacterial usage by PAL category across Australian Principal Referral hospitals (de-identified), 2019**
